# Prevalence of Denture-Related Stomatitis in Edentulous Patients at a Tertiary Dental Teaching Hospital

**DOI:** 10.3389/froh.2021.772679

**Published:** 2021-12-01

**Authors:** Razia Zulfikar Adam, Faheema Kimmie-Dhansay

**Affiliations:** ^1^Department of Restorative Dentistry, Faculty of Dentistry, University of the Western Cape, Cape Town, South Africa; ^2^Division of Research and Postgraduate Studies, Faculty of Dentistry, University of the Western Cape, Cape Town, South Africa

**Keywords:** candidiasis, edentulism, complete dentures, prevalence, denture related stomatitis

## Abstract

Oral mucosal lesions can be uncomfortable and can result in a poorer oral health related quality of life. This can be seen especially in edentulous patients who are mostly elderly and have comorbid diseases such as Diabetes Mellitus, which can impair their ability to withstand oral infections. In South Africa, one of the most unequal countries in the world, almost 50% of the population is edentulous and this prevalence increases as age increases. The aim of this cross-sectional study was to determine the prevalence of denture-related stomatitis in subjects who presented to a tertiary institution in Cape Town, South Africa for new complete dentures and to determine the risk indicators associated with it. Three hundred and ninety-six folders of participants who received complete dentures during the period 2014–2019 were included in this study. Categorical data was displayed as frequencies and percentages and a multiple adjusted logistic regression was used to determine associations between Candida and certain risk indicators. The prevalence of denture-related stomatitis was 25.76% (*n* = 102). Almost 75% (*n* = 225) females and 72.63% (*n* = 69) males had no denture-related stomatitis. The most common site for candidiasis in this population was the palate and tonsillar area (40.2%, *n* = 41) and the least common site was the upper ridge (2.94%, *n* = 3). Candidiasis, in edentulous patients are highly prevalent in this population and more needs to be done to prevent it.

## Introduction

Worldwide people are living longer. Healthcare improvements in the last century have led to an increasingly elderly population. The World Health Organization (WHO) estimates that by 2050 the world's population of 60 years and older will total 2 billion [1]. It is known that aging is coupled with the presence of disease which reduces the quality of life and impacts life expectancy.

The Global Burden of Disease (GBD) 2015 study, calculates that around “3.5 billion people worldwide live with dental conditions, predominantly untreated dental caries in the deciduous and permanent dentitions, severe periodontal disease, edentulism (complete tooth loss), and severe tooth loss (having between 1 and 9 remaining teeth)” [2]. In addition to this, 80% of older people will be living in low- and middle-income countries by 2050.

South Africa is reported to be one of the most unequal countries in the world with a Gini index of 0.65 [3]. The inequality of the country leads to unequal access to medical care where only the minority of the population has access to comprehensive medical care. The vast majority of the population is unfortunately only afforded care to the overburdened and under-funded health care system. The health care system in South Africa thus only provides essential dental services such as exodontia and limited preventative care. In South Africa, 12.6% of the population aged 35–44 years was completely edentulous according to the 1988/89 National Oral Health Survey [4]. More recently in a study conducted in Bellville South, Cape Town, South Africa, the edentulous prevalence was close to 50% [5]. The overburdened healthcare system and increased edentulous rates results in significant challenges for oral healthcare delivery, to an increasingly aged population with declining oral health. As the population ages, the oral diseases become more relevant concerning their local and systemic impact, which can have profound implications for healthcare provision. Together with a lack of adequate preventative oral health care services, extractions have been the favored treatment modality increasing the number of edentulous patients in South Africa.

Numerous studies conducted in different countries across the world have reported on the risk factors of edentulism. Kailembo et al. described edentulism patterns in China, India, Ghana and South Africa using data from the World Health Organization global AGEing study and adult health (SAGE) Wave 1 (2007–2010) [6]. Risk factors to edentulism include smoking, alcohol consumption and poor nutrition. Studies investigating the relationship between gender and edentulism revealed conflicting results although observational studies suggest that females were more likely to be edentulous. Differences can also be seen across rural and urban settings in countries as access to refined food or oral healthcare may be determinants.

Consequences of tooth loss and eventually edentulism include resorption of alveolar bone, changes in talking, aesthetics, chewing, and digestion. Besides, there is evidence linking edentulism with an impaired quality of life which in turn affects morbidity and mortality [7, 8]. Oliveira et al. reported that edentulism- free life expectancy among older Brazilian adults decreased with age and increased over the study period [7]. Sex and education inequalities were also observed.

Edentulous patients are also more likely to suffer from oral mucosal lesions related to the wearing of removable prosthesis [9–14]. The most commonly seen denture-related oral mucosal lesions (DOML) are stomatitis, hyperplasia, angular cheilitis and traumatic ulcers [9–14]. DOML was also seen more frequently in patients who wore complete dentures as opposed to removable partial dentures [9]. This could be explained by the presence of Candida, mechanical issues, long term wear and greater mucosal coverage by the denture base area [14]. Few studies reported on the duration of dentition wear and the relationship with the presence of DOML [9, 10, 12, 14]. Patients who were denture wearers for longer were more likely to have DOML.

It is important to routinely collect epidemiological information to evaluate the needs of a population. No recent data is available for the prevalence of denture-related stomatitis in a South African population. The purpose of the study was to determine the prevalence of denture-related stomatitis (DRS) in complete denture wearers and the risk factors associated with it.

## Methodology

A retrospective records-based study design was conducted in student dental clinics at a tertiary hospital in Cape Town, South Africa. Information was collected from patient files who received complete dentures in the period 2014–2019. Data collected included the patient's age, gender, medical conditions, denture experience, presence of oral candidiasis, site of lesion, and treatment for the condition. Patients were further classified using the Uniform Patient Fee Schedule (UPFS). Patients treated in government hospitals are charged according to the UPFS, which sets a tariff according to the level of hospital providing the treatment, category of medical staff providing the treatment and the income of the patient. The categories are:Full subsidization for participants who are pensioners or unemployed. Any participant who earns less than ZAR 250,000 per individual or ZAR 350,000 per family per year, will be classified as “other” income category. Patients usually treated in these clinics are examined by students and academic teaching staff. World Health Organization guidelines are used to diagnose oral mucosal lesions. Denture-related stomatitis lesions were classified according to Newton's classification: Type 1: scattered spot areas of the palatine mucosa inflammation dispersed throughout the normal mucosa; type 2: palatal mucosa presenting as generalized inflammation in the area covered by the prosthesis; type 3: hyperaemic palatal mucosa presenting nodular appearance [15]. All records were anonymised. Ethics approval was granted by the Biomedical Research Committee of the University of the Western Cape (BM20/5/14). Only patients who were completely edentulous, diagnosed with DRS and received a set of complete dentures were included in the study. Sample size was not determined as all the subjects who received complete dentures during 2014–2019 were included.

### Statistical Analysis

Continuous data was presented as means and standard deviations if normally distributed. Categorical data was presented as frequencies and percentages and chi squared tests were used to determine associations between two categorical variables. A simple logistic regression was used to determine associations where there were two variables. An adjusted multiple logistic regression with backward elimination was used to determine if there was any association with DRS and other variables such as sex, income category, hypertension, diabetes, mental condition, Tuberculosis, smoking history, history of sleeping with denture, denture cleaning practices, age of the subject, and age of existing denture. Cutoff point for inclusion of variables was set at *p* < 0.1. All data was analyzed with StataCorp 2019. Stata Statistical Software: Release 16. College Station, TX: StataCorp LLC. All tests for bivariate analysis were deemed statistically significant at *p* < 0.05.

## Results

### Demographic Characteristics

**Figure 1:**
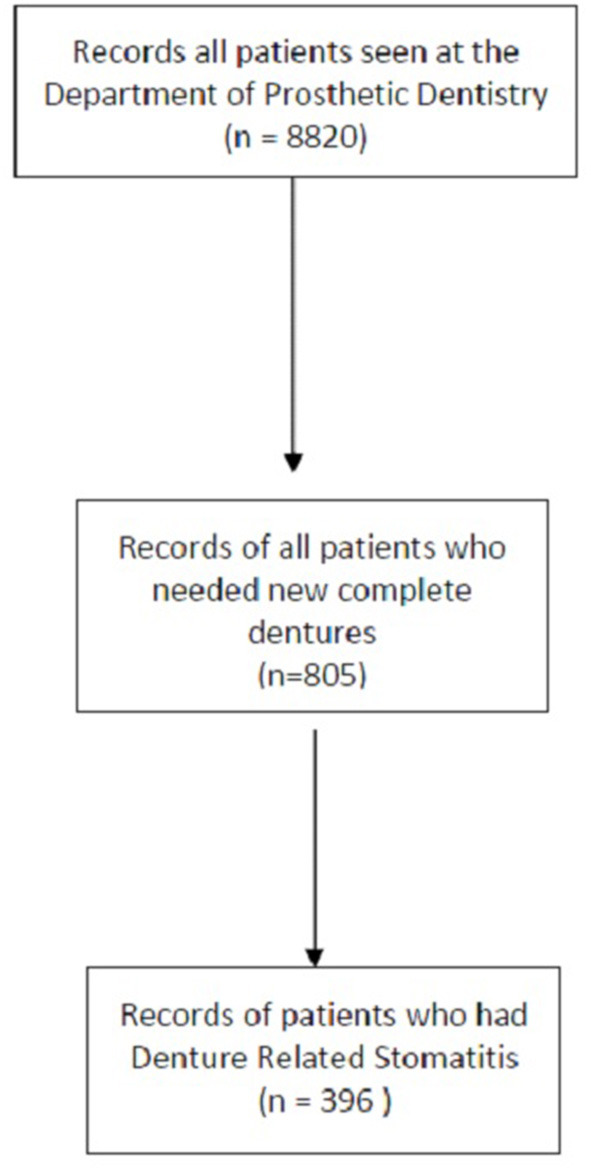
Summary of sample population.

Information from 805 folders was examined (Figure 1). Three hundred and ninety-six participants had an existing denture and was presenting for a new denture at the tertiary institution. The median age of the participants was 64.3 [58.9–70.15]. The percentage of females and males were 76.01% (*n* = 301) and 23.99% (*n* = 95), respectively. Seventy-eight percent (*n* = 311) of the patients treated were fully subsidized patients and received a social grant. The most frequent systemic disease was hypertension 62.03% (*n* = 245) followed by diabetes mellitus, 26.21% (*n* = 103), mental disorder, 0.8% (*n* = 3), and Tuberculosis 0.3% (*n* = 1). More than a quarter of the patients (*n* = 114) were smokers (30.24%), as seen in Table 1.

**Table 1:** Distribution of demographic data of the patients.

		***n* (%)**	**No denture related stomatitis *n* (%)**	**Denture related stomatitis**	***P*-value**
Total		396 (100.0)	294 (74.2)	102 (25.8)	
Sex	Female	301 (76.0)	225 (76.5)	76 (74.5)	
	Male	95 (24.0)	69 (23.5)	26 (25.5)	0.681
UPFS category	H0	311 (78.5)	233 (79.3)	78 (76.5)	
	Other	85 (21.5)	61 (20.7)	24 (23.5)	0.556
Hypertension	No	150 (38.0)	114 (38.9)	36 (35.3)	
	Yes	245 (62.0)	179 (61.1)	66 (64.7)	
	Not recorded	1 (0.25)	1 (0.34)	0 (0)	0.517
Diabetes	No	290 (73.8)	217 (74.3)	73 (72.3)	
	Yes	103 (26.2)	75 (25.7)	28 (27.7)	
	Not recorded	3 (0.76)	2 (0.68)	1 (0.98)	0.688
Mental condition	None	387 (99.2)	289 (99.7)	98 (98.0)	
	Yes	3 (0.8)	1 (0.3)	2 (2.0)	
	Not recorded	6 (1.52)	4 (1.36)	2 (1.96)	0.102
TB	None	389 (99.7)	289 (99.7)	100 (100.0)	
	Yes	1 (0.3)	1 (0.3)	0 (0.0)	
	Not recorded	6 (1.52)	4 (1.36)	2 (1.96)	0.556
Smoker	No	263 (69.8)	195 (69.6)	68 (70.1)	
	Yes	114 (30.2)	85 (30.4)	29 (29.9)	
	Not recorded	19 (4.79)	14 (4.76)	5 (4.90)	0.932
Sleeps with denture	None	140 (58.3)	120 (74.1)	20 (25.6)	
	Yes	100 (41.7)	42 (25.9)	58 (74.4)	
	Not recorded	156 (39.39)	132 (44.90)	24 (23.53)	<0.001^*^
Cleans Denture	None	210 (71.4)	65 (63.7)	275 (69.4)	
	Yes	84 (28.6)	37 (36.3	121 (30.6)	0.1456

* *Statistically significant*.

### Risk Factors

Of these participants, 21.42% (*n* = 108) had never owned a complete set of dentures before. The median age of dentures was 10 [6–20] years. 41.67% (*n* = 100) reported sleeping with their dentures and 25.76% (*n* = 102) was found to have some form of oral candidiasis. Majority of the participants did not report cleaning their dentures (30.56%, *n* = 121). Participants commonly used toothpaste, (27.27% (*n* = 108). Sex, income category, HT, DM, mental condition, TB, smoking status, denture hygiene practice had no impact on the presence of DRS. However, increased age of the denture and the age of the subject had an impact on the presence of DRS.

### Denture-Related Stomatitis and Risk Indicators

More than 76.5% (*n* = 225) females and 72.63% (*n* = 69) males had no stomatitis. Just over 25% (*n* = 102) had a diagnosis of oral candidiasis. The prevalence of DRS in female patients (74.51%, *n* = 76) was higher than in men (25.49%, *n* = 26; *p* = 0.68). In the relationships between systemic disease (hypertension, tuberculosis, diabetes, mental health issues), smoking and the presence of oral candidiasis, no significant associations (*p* > 0.05) was observed. 76.47% (*n* = 78) of patients who were receiving social grants presented with oral candidiasis.

Using a simple logistic regression, patients who had a practice of sleeping with dentures at night were more likely to develop oral candidiasis (*p* < 0.001; OR: 8.286; 95% CI: 4.47–15.37) and the age of the denture determined the likelihood of presenting with oral candidiasis (*p* = 0.026; OR: 1.03; CI: 1.003–1.048). When a multiple logistic regression was used, when backwards elimination, only participants who slept with their denture, 8.56 (4.565–16.015) was statistically significant (*p* < 0.0001^*^), Table 2.

**Table 2:** Logistic regression -Denture related stomatitis.

	**Model 1 (Simple logistic regression)**			**Model 2 (Adjusted multiple logistic regression)**			**Model 2 (Final adjusted multiple logistic regression)**		
	**Unadjusted OR**	**95% CI**	***P*-value**	**Adjusted OR**	**95% CI**	***P*-value**	**Final adjusted OR**	**95% CI**	***P*-value**
Gender = f	1.000			1.000					
Gender = m	1.116	0.663 1.878	0.681	1.115	0.5534 2.4109	0.701			
Category = H0	1.000			1.000					
Category = Other	1.175	0.687 2.012	0.556	1.267	0.5359 2.997	0.590			
Hypertension = n	1.000			1.000					
Hypertension = y	1.168	0.730 1.866	0.517	1.295	0.6362 2.6376	0.475			
Diabetes = n	1.000			1.000					
Diabetes = y	1.110	0.667 1.846	0.688	1.457	0.6908 3.0721	0.323			
Smoker = n	1.022	0.618 1.692	0.932	1.396	0.6715 2.9007				
Smoker = y	1.000			1.000		0.372			
Clean = does not clean denture	1.000			1.000					
Clean = cleans denture	1.423	0.884 2.292	0.147	1.493	0.7643 2.9164	0.241			
Sleeps with denture = n	1.000			1.000					
Sleeps with denture = y	8.286	4.467 15.370	<0.001^*^	10.342	5.229 20.4517	<0.0001^*^	8.56	4.565 16.015	<0.0001^*^
Denture age (years)	1.025	1.003 1.048	<0.026^*^	1.030	0.997 1.064	0.078	1.027	0.996 1.059	0.085
Age	1.007	0.980 1.035	0.630	1.027	0.982 1.074	0.239			
Intercept				0.008	0.000 0.168	0.002^*^	0.112	0.057 0.220	<0.001^*^

*OR, Odds ratio; 95% C.I., 95% confidence interval*;

* *Statistically significant*.

### Location of DRS Lesions

The most common site for oral mucosal lesions in this population was the palate and tonsillar area (40.2%, *n* = 41) and the least common site was the upper ridge (2.94%, *n* = 3).

## Discussion

There have been many reports on the oral hygiene habits and the prevalence of OMLs in adults and the elderly or institutionalized [16, 17]. However, there are few studies on the oral and general health of complete denture wearers in the South African population [18]. The purpose of this research was to explore the prevalence of denture-related stomatitis and risk factors at a tertiary hospital in Cape Town, South Africa.

Results from our study show a total prevalence of DRS of 25.8% and DOML of 17%. These results are slightly different to Du Toit & Claassen, who conducted a similar study amongst denture wearers at the same site [18]. However, internationally a higher prevalence was recorded. Brantes et al. reported a prevalence of 78%, Perić et al. reported 38.8% and Wang et al. - 42.7% [9, 13, 19]. The variation in prevalence studies, could be attributed to the difference in study designs, populations, settings and whether all removable prostheses were included.

In contrast to other studies [9, 12, 13], the authors found no significant relationship with gender. However, age was an important risk factor. Almost all the patients were 50 years and older and this could explain the risk in this particular study. Patients who are older are also more likely to suffer from a systemic disease such as diabetes which could predispose them to the development of oral candidiasis. Older patients may also experience changes in their mouth and saliva due to age. Elderly patients often experience xerostomia and the hyposalivation may result a constant dryness which favors the proliferation of bacteria.

Patients in this study had worn the same set of dentures for an average of 10 years. This poses a significant risk to the development of DRS. The deterioration of dentures over a period of use results in wear of the acrylic, irregular surface roughness, and the possibility of cracks and fractures. This will increase the development of fibrous hyperplasia, denture associated stomatitis, angular cheilitis, and traumatic ulcers. Usually after 2 years, acrylic teeth exhibit signs of wear and signs of an altered vertical dimension in patients may be present. In this study, the majority of the patients were subsidized and receiving a social grant. In the Western Cape, this tertiary level student run dental clinic is the only public health facility providing dentures for the public and as a result there is a lengthy waiting list. In addition, the high cost of making a denture privately prevents patients replacing them sooner. The site of denture-related stomatitis was not reported on in similar studies [18, 20] and this could be because denture-related stomatitis is described as affecting the maxilla palatal area.

There is no gold standard of care for complete dentures. A recent systematic review evaluated chemical cleaning methods to eliminate Candida from dentures [21]. The authors reported that there was weak evidence for the use of chemical cleaning methods although chlorhexidine and chlorine dioxide showed a reduction in Candida CFUs. A recent study found that 0.25% sodium hypochlorite and 0.15% Triclosan treatments significantly reduced Gram-negative microorganisms in a randomized control trial [22]. Chemical denture cleaning protocols that are locally appropriate must be shared with patients and included in the dental curricula. Included in these care protocols, patients must be motivated to remove dentures prior to sleeping.

## Conclusion

The present study demonstrated that among people wearing complete dentures, who attend the Dental Faculty, the prevalence of denture stomatitis is high. The age of the denture and whether the subject slept with the denture resulted in a higher prevalence of denture stomatitis. Patient education around denture hygiene and storage is necessary to avoid denture-related stomatitis.

## Data Availability Statement

The datasets presented in this study can be found in online repositories. The names of the repository/repositories and accession number(s) can be found at: Adam et al. (2021). Prevalence of oral mucosal lesions in edentulous patients at a tertiary dental teaching hospital [Data set]. Zenodo. https://doi.org/10.5281/zenodo.5494735.

## Ethics Statement

The studies involving human participants were reviewed and approved by Biomedical Research Committee of the University of the Western Cape (BM20/5/14). The patients/participants provided their written informed consent to participate in this study.

## Author Contributions

RA was responsible for the conceptualization, methodology, investigation, data curation, writing original draft, writing—review and editing, visualization, funding acquisition, and supervision. FK-D was responsible for writing—review and editing, visualization, and formal analysis. All authors contributed to the article and approved the submitted version.

## Funding

This research was funded by the National Research Foundation as part of the BAAP programme for RA (BAAP190416431001).

## Conflict of Interest

The authors declare that the research was conducted in the absence of any commercial or financial relationships that could be construed as a potential conflict of interest.

## Publisher's Note

All claims expressed in this article are solely those of the authors and do not necessarily represent those of their affiliated organizations, or those of the publisher, the editors and the reviewers. Any product that may be evaluated in this article, or claim that may be made by its manufacturer, is not guaranteed or endorsed by the publisher.
